# High-throughput single nucleotide polymorphism genotyping using nanofluidic Dynamic Arrays

**DOI:** 10.1186/1471-2164-10-561

**Published:** 2009-11-28

**Authors:** Jun Wang, Min Lin, Andrew Crenshaw, Amy Hutchinson, Belynda Hicks, Meredith Yeager, Sonja Berndt, Wen-Yi Huang, Richard B Hayes, Stephen J Chanock, Robert C Jones, Ramesh Ramakrishnan

**Affiliations:** 1 Fluidigm Corporation, South San Francisco, CA, USA; 2 Core Genotyping Facility, National Cancer Institute, SAIC-Frederick, Inc, Advanced Technology Program, NCI-FCRDC, Frederick, MD, USA; 3 Division of Cancer Epidemiology and Genetics, National Cancer Institute, NIH, Bethesda, MD, USA

## Abstract

**Background:**

Single nucleotide polymorphisms (SNPs) have emerged as *the *genetic marker of choice for mapping disease loci and candidate gene association studies, because of their high density and relatively even distribution in the human genomes. There is a need for systems allowing medium multiplexing (ten to hundreds of SNPs) with high throughput, which can efficiently and cost-effectively generate genotypes for a very large sample set (thousands of individuals). Methods that are flexible, fast, accurate and cost-effective are urgently needed. This is also important for those who work on high throughput genotyping in non-model systems where off-the-shelf assays are not available and a flexible platform is needed.

**Results:**

We demonstrate the use of a nanofluidic Integrated Fluidic Circuit (IFC) - based genotyping system for medium-throughput multiplexing known as the Dynamic Array, by genotyping 994 individual human DNA samples on 47 different SNP assays, using nanoliter volumes of reagents. Call rates of greater than 99.5% and call accuracies of greater than 99.8% were achieved from our study, which demonstrates that this is a formidable genotyping platform. The experimental set up is very simple, with a time-to-result for each sample of about 3 hours.

**Conclusion:**

Our results demonstrate that the Dynamic Array is an excellent genotyping system for medium-throughput multiplexing (30-300 SNPs), which is simple to use and combines rapid throughput with excellent call rates, high concordance and low cost. The exceptional call rates and call accuracy obtained may be of particular interest to those working on validation and replication of genome- wide- association (GWA) studies.

## Background

Single nucleotide polymorphisms (SNPs) have emerged as *the *genetic marker of choice for mapping disease loci and candidate gene association studies, because of their high density and relatively even distribution in the human genomes [1]. Both the International Human Genome Sequencing Project and the International HapMap Projects have generated large amounts of data on the location, quantity, type, and frequency of genetic variants, in particular of SNPs, in the human genome [2-7]. The determination of genome-wide linkage disequilibrium (LD) patterns through the HapMap project has enabled the selection of markers for efficient genome-wide association (GWA) studies in human samples[8]. Recent technological advances in ultra high-throughput genotyping platforms potentially permit the parallel analysis of millions of SNPs with a significant reduction in genotyping price, making GWA studies a reality [9-13].

The impact of this approach is readily visible, since over 296 publications have been collected in the last three years in the National Cancer Institute (NCI)-National Human Genome Research Institute (NHGRI)'s catalog of published genome-wide association studies [14].

Many challenges still exist after this first wave of GWA studies, and one of them is the validation and replication of the positive hits from the GWA screening. A multistage approach has been suggested to increase GWA efficiency with proper validation steps [8]. A large number of individuals are usually required to be genotyped on a small subset of candidates SNPs. Many SNP genotyping methods have been developed on varieties of platforms in the past decade [15,16], with several technologies using solid-phase-mediated detection being able to genotype thousands of sites simultaneously, such as the GeneChip array and the BeadArray [17-19]. These methods have provided ultra multiplex and high-throughput genotyping but are not cost effective for genotyping a large number of samples for a modest number of SNPs. Homogenous detection methods such as TaqMan^® ^[20] and molecular beacon -based [21] approaches provide uniplex reactions, and are readily applied to a large number of samples but multiplexing may be difficult. Between these two (ultra-high multiplexing and low/no multiplexing) methods there is a need for systems allowing medium multiplexing (ten to hundreds of SNPs) with high throughput, which can efficiently and cost-effectively generate genotypes for a very large sample set (thousands of individuals). Therefore, alternative methods that are flexible, fast, accurate and cost-effective are urgently needed. This is also important for those who work on high throughput genotyping in non-model systems where off-the-shelf assays are not available and a flexible platform is needed.

We have developed a nanofluidic platform to meet the needs of medium multiplex high throughput SNP genotyping and demonstrate call rates of >99% and accuracy rates of >99.8% under uniform assay conditions, on human samples.

## Results and Discussion

### Chip Architecture

**Figure 1:**
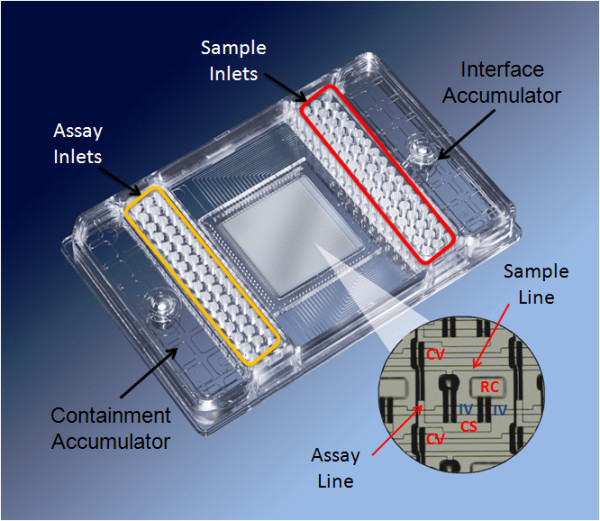
**A 48.48CS dynamic array showing the position of the sample inlets and the assay inlets**.

The chip used in this study, the 48.48CS dynamic array, is mounted on a plastic carrier with interface and containment accumulators and 48 sample inlets and 48 assay inlets, with the dimensions of the inlets and the size of the plate conforming to the standards set by the Society for Biomolecular Sciences (SBS format). The chip has the ability to test 48 samples with 48 SNP assays. These combine in pair wise fashion to produce 2304 reaction chambers, in a final volume of 6.75 nl (Figure 1). A similar chip has been described previously by Spurgeon et.al (2008) [22]. The integrated fluidic circuit (IFC) is a network of fluid lines, NanoFlex™ valves and chambers. The NanoFlex™ valves are made of an elastomeric material which deflects under pressure to create a tight seal and are used to regulate the flow of liquids in the IFC. Prior to loading, the chip is primed using the NanoFlex™ 4-IFC Controller which pressurizes the control lines and closes the interface valves. Individual samples are pipetted into the sample inlets and genotyping TaqMan^® ^assays are pipetted into the detector inlets. The chip is then placed back on the NanoFlex™ 4-IFC Controller for loading and mixing. During this process, pressure is applied to the fluid in the sample inlets and the fluid is pushed into the sample fluid lines. At the same time the fluid in the assay inlets is pushed into the assay fluid lines. Mixing of the two fluids is prevented by the closed interface valve.

This chip (Figure 1) utilizes a design known as the carry-over slug (CS) design, providing precise metering of fluid volumes and efficient mixing of the metered volumes. A first solution (comprising of a specific TaqMan^® ^assay) is introduced into a segment of a flow channel in fluidic communication with a reaction chamber. A second solution (the sample mix) is flowed through the segment so that the first (assay) solution is displaced into the reaction chamber, and a volume of the second (sample) solution enters the chamber. The chamber is then isolated and reactions within the chamber can be initiated by thermal cycling. This load-mix process takes approximately 45 minutes. After loading and mixing is complete, the chip is placed on a standalone PCR thermal cycler for PCR. The volumes involved in performing genotyping on the Dynamic Array significantly reduces the genotyping reagent consumption and labor cost (see Table 1).

**Table 1:** Comparison of Genotyping Reagent Consumption

	48.48 array	384-well plate	Fold saving
Total number of reactions	2304	2304	
Number of chips/plates	1	6	
Volume of each reaction	6.75 nL	10 μL	
2× Mastermix (μL)	120*	11520	96
10× Primer/Probe Mix (μL)	24*	2304	96
Number of pipetting steps/tips	96	4608	48

* On the Dynamic Array using 5 μL reaction mixes for 48 samples at a time

**Figure 2:**
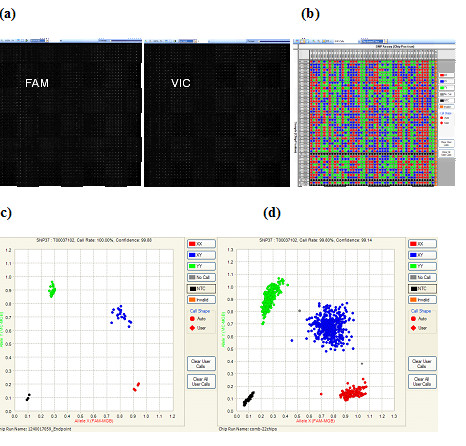
**SNP Genotyping data analysis**. The Fluidigm SNP Genotyping Analysis software automatically analyzes the end-point image of a genotyping chip run and generates genotyping calls for each sample. **(a)**. Raw image from a 48.48CS chip run in both FAM and VIC fluorescent channels. **(b)**. The software generated call map view of the genotyping calls for each of the 2304 reaction chambers. **(c) **Software generated scatter plot for 48 samples in one SNP assay with genotype calls automatically. Four different color coded, 3 genotypes plus negative controls (NTC, black dots) are observed **(d) **Genotyping scatter plot of samples from 22 chip runs.

Once PCR is complete, endpoint fluorescent image data is acquired on the BioMark™ System for Genetic Analysis and data is analyzed using the Fluidigm SNP Genotyping Analysis software, to obtain genotype calls. Figure 2a shows the raw image from a chip run in both FAM (excitation and emission peaks at 495 and 520 nm respectively) and VIC (excitation and emission peaks at 538 and 554 nm respectively) fluorescent channels. Figure 2b shows a computer generated image of the genotype calls for each of the 2304 reaction chambers. Each column (vertical direction) represents data from one assay that correlated to the SNP genotyping assay loaded from each assayinlet.

Each row represents data from one DNA sample loaded from each sample inlet. In brief, the genotyping software calculates the fluorescent signals from both FAM and VIC channels and plots each sample on a scatter plot using the FAM relative intensity (to ROX background) on the X-axis, and VIC relative intensity on the Y-axis. We then used a k-means clustering algorithm based on nearest-centroid sorting to automatically classify samples into four genotype groups, or three if the rare homozygous allele is not present in a particular chip run. Figure 2c shows a genotyping scatter plot with genotype calls automatically assigned by the analysis software with 4 different colors coded, one for each genotype plus one NTC (black dots). The software is capable of analyzing multiple chip runs simultaneously, and up to 22 chip runs were analyzed together in this study (Figure 2d).

### Human SNP Genotyping

**Figure 3:**
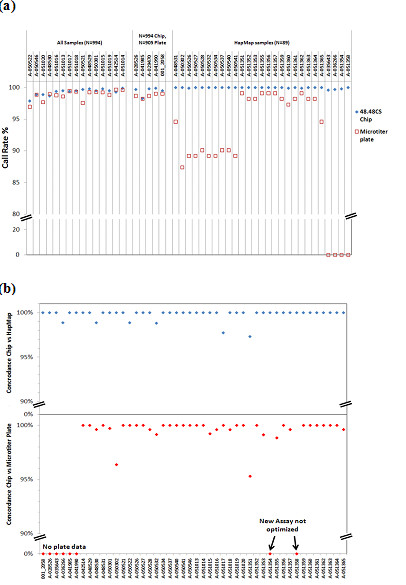
**Comparison of genotyping results from 48.48CS dynamic array with microtiter plates run on Applied Biosystems 7900 HT**. **(a) **Call rate comparison; **(b) **Concordance/Accuracy with HapMap results.

We utilized this nanofluidic system to genotype SNPs in human genomic DNA samples. The DNA screened included 905 DNA samples extracted from blood from the Prostate, Lung, Colon, and Ovarian Cancer (PLCO) Screening Trial [23], and 89 HapMap samples extracted from cell lines. All samples were double-blinded, and were genotyped on 22 dynamic arrays. Chips were thermal cycled using standard conditions (see materials and methods), and the end-point fluorescence values were measured on the BioMark™ system. A total of 2 μl of each DNA sample (50 ng) was used in a 5 μl sample mix to genotype 47 SNP assays and one non-reagent control. Each chip only needs a total of 96 liquid-transfer (pipetting) steps (48 for the sample and 48 for the assay mixes), which is considerably less pipetting than required to set up 6 conventional 384 well plates (4608 pipetting, 2304 × samples plus 2304 × assays) with the same genotyping output. We manually set up 8 chip runs on each working day with a throughput of about 18,000 genotypes per day, without using robotic stations. The endpoint genotype data were analyzed using the Fluidigm SNP Genotyping Analysis software (Figure 2). We achieved a call rate of greater than 99.1% from the 994 genotyped samples on 47 SNPs. In comparison, we have also used an Applied Biosystems 7900 HT with standard microtiter plates and TaqMan^® ^genotyping methodologies to genotype the 905 PLCO samples on 20 SNPs and the 89 HapMap samples on 38 SNPs, and obtained a lower call rate at 98.5% with almost every SNP in comparison to the results obtained on the Fluidigm dynamic array (Figure 3a, Table 2). We have estimated the genotyping concordance between the results from the 48.48CS chip and the microtiter plates run on the Applied Biosystems 7900 HT (Figure 3b) system. Among the 20 SNPs genotyped on 905 PLCO samples, all SNPs gave ≥ 99.5% concordance, except for one with 98.8%. Among the 89 HapMap samples genotyped by both platforms, all SNPs except three had concordance rates of 99% to 100% (Figure 3b).

**Table 2:** Completion Rates comparing the Fluidigm platform with standard TaqMan chemistry (CGF)

Assay	Fluidigm Completion Rate	95% CI lower	95% CI upper	N	CGF Completion Rate	95% CI lower	95% CI upper	M	Completion rate based on
A-050522	97.89	97.00	98.78	995	96.95	95.88	98.02	995	All Samples (N = M = 995)
A-050546	98.89	98.24	99.54	995	98.85	98.19	99.51	995	All Samples (N = M = 995)
A-051020	98.89	98.24	99.54	995	97.66	96.72	98.60	995	All Samples (N = M = 995)
A-048530	98.69	97.99	99.40	995	98.96	98.33	99.59	995	All Samples (N = M = 995)
A-051016	99.40	98.92	99.88	995	98.78	98.10	99.46	995	All Samples (N = M = 995)
A-028526	99.70	99.36	100.00	995	98.66	97.91	99.41	905	N = 995 Fluidigm M = 905 CGF (90 HapMap excluded)
A-041985	98.29	97.49	99.10	995	98.22	97.36	99.08	905	N = 995 Fluidigm M = 905 CGF (90 HapMap excluded)
A-051013	99.50	99.06	99.94	995	98.59	97.86	99.32	995	All Samples (N = M = 995)
A-051017	99.40	98.92	99.88	995	99.41	98.93	99.89	995	All Samples (N = M = 995)
A-051018	99.50	99.06	99.94	995	99.32	98.81	99.83	995	All Samples (N = M = 995)
A-029470	99.80	99.52	100.00	995	98.66	97.91	99.41	905	N = 995 Fluidigm M = 905 CGF (90 HapMap excluded)
A-050521	99.70	99.36	100.00	995	97.55	96.59	98.51	995	All Samples (N = M = 995)
A-048529	99.80	99.52	100.00	995	99.25	98.71	99.79	995	All Samples (N = M = 995)
A-041990	99.90	99.70	100.00	995	99	98.35	99.65	905	N = 995 Fluidigm M = 905 CGF (90 HapMap excluded)
001_2058	99.50	99.06	99.94	995	99	98.35	99.65	905	N = 995 Fluidigm M = 905 CGF (90 HapMap excluded)
A-050301	99.50	99.06	99.94	995	99.28	98.75	99.81	995	All Samples (N = M = 995)
A-051015	99.80	99.52	100.00	995	99.22	98.67	99.77	995	All Samples (N = M = 995)
A-051019	99.50	99.06	99.94	995	98.82	98.15	99.49	995	All Samples (N = M = 995)
A-042514	99.30	98.78	99.82	995	99.65	99.28	100.00	995	All Samples (N = M = 995)
A-051014	99.90	99.70	100.00	995	99.61	99.22	100.00	995	All Samples (N = M = 995)
A-035643	99.60	98.29	100.00	90	N.D.	N.D	N.D.	N.D.	N = 90 Fluidigm HapMap
A-036266	99.70	98.57	100.00	90	N.D.	N.D.	N.D.	N.D.	N = 90 Fluidigm HapMap
A-048531	100.00	100.00	100.00	90	94.59	89.92	99.26	90	N = 90 Fluidigm HapMap and M = 90 CGF HapMap
A-050302	100.00	100.00	100.00	90	87.39	80.53	94.25	90	N = 90 Fluidigm HapMap and M = 90 CGF HapMap
A-050526	99.90	99.24	100.00	90	89.19	82.77	95.61	90	N = 90 Fluidigm HapMap and M = 90 CGF HapMap
A-050527	100.00	100.00	100.00	90	89.19	82.77	95.61	90	N = 90 Fluidigm HapMap and M = 90 CGF HapMap
A-050528	100.00	100.00	100.00	90	90.09	83.92	96.26	90	N = 90 Fluidigm HapMap and M = 90 CGF HapMap
A-050532	100.00	100.00	100.00	90	89.19	82.77	95.61	90	N = 90 Fluidigm HapMap and M = 90 CGF HapMap
A-050534	100.00	100.00	100.00	90	89.19	82.77	95.61	90	N = 90 Fluidigm HapMap and M = 90 CGF HapMap
A-050537	100.00	100.00	100.00	90	90.09	83.92	96.26	90	N = 90 Fluidigm HapMap and M = 90 CGF HapMap
A-050540	100.00	100.00	100.00	90	90.09	83.92	96.26	90	N = 90 Fluidigm HapMap and M = 90 CGF HapMap
A-050541	100.00	100.00	100.00	90	89.19	82.77	95.61	90	N = 90 Fluidigm HapMap and M = 90 CGF HapMap
A-051351	100.00	100.00	100.00	90	99.1	97.15	100.00	90	N = 90 Fluidigm HapMap and M = 90 CGF HapMap
A-051352	100.00	100.00	100.00	90	98.2	95.45	100.00	90	N = 90 Fluidigm HapMap and M = 90 CGF HapMap
A-051353	100.00	100.00	100.00	90	98.2	95.45	100.00	90	N = 90 Fluidigm HapMap and M = 90 CGF HapMap
A-051354	99.80	98.87	100.00	90	N.D.	N.D.	N.D.	N.D.	N = 90 Fluidigm HapMap
A-051355	100.00	100.00	100.00	90	99.1	97.15	100.00	90	N = 90 Fluidigm HapMap and M = 90 CGF HapMap
A-051356	100.00	100.00	100.00	90	99.1	97.15	100.00	90	N = 90 Fluidigm HapMap and M = 90 CGF HapMap
A-051357	100.00	100.00	100.00	90	99.1	97.15	100.00	90	N = 90 Fluidigm HapMap and M = 90 CGF HapMap
A-051358	100.00	100.00	100.00	90	N.D.	N.D.	N.D.	N.D.	N = 90 Fluidigm HapMap
A-051359	100.00	100.00	100.00	90	98.2	95.45	100.00	90	N = 90 Fluidigm HapMap and M = 90 CGF HapMap
A-051360	99.90	99.24	100.00	90	97.3	93.95	100.00	90	N = 90 Fluidigm HapMap and M = 90 CGF HapMap
A-051361	100.00	100.00	100.00	90	98.2	95.45	100.00	90	N = 90 Fluidigm HapMap and M = 90 CGF HapMap
A-051362	99.90	99.24	100.00	90	99.1	97.15	100.00	90	N = 90 Fluidigm HapMap and M = 90 CGF HapMap
A-051363	100.00	100.00	100.00	90	98.2	95.45	100.00	90	N = 90 Fluidigm HapMap and M = 90 CGF HapMap
A-051364	100.00	100.00	100.00	90	98.2	95.45	100.00	90	N = 90 Fluidigm HapMap and M = 90 CGF HapMap
A-051365	100.00	100.00	100.00	90	94.59	89.92	99.26	90	N = 90 Fluidigm HapMap and M = 90 CGF HapMap

We next estimated the genotyping accuracy after unblinding the samples. The genotyping results obtained from the 89 HapMap DNA samples were compared with the corresponding data in the dbSNP database http://www.ncbi.nlm.nih.gov/projects/SNP/. We achieved a call rate of 99.8% for all samples across 47 genotyped SNPs (4183 genotypes) using the BioMark™ System. A total of 3950 genotypes of the tested SNPs were found in the dbSNP database for these HapMap samples; our genotyping results matched 3943 with only 7 mismatches, resulting in a 99.82% concordance. Almost all of the SNPs except for six gave 100% concordance (Figure 3b). We also determined the reproducibility (concordance) of our system. As mentioned earlier, some of the 905 PLCO samples had been provided in (blind) replicates. The replicates comprised a total of 130 samples, and included 54 duplicates (108 samples), 6 triplicates (18 samples) and 1 quadruplicate (4 samples) set, resulting in 65 pair-wise comparisons. Of the 65 pair wise comparisons 55 pairs exhibited 100% concordance for 47 SNPs. Of the discordant pairs of samples: 8 pairs had a single discordant result; 1 pair had two discordant results and 1 pair had three discordant results. Of the 47 SNP assays, 43 have no discordance. Of the discordant assays: 1 assay exhibited a single discordance; 2 assays exhibited two discordances and 2 assays exhibited four discordances. The total reproducibility rate is > 99.78%.

**Figure 4:**
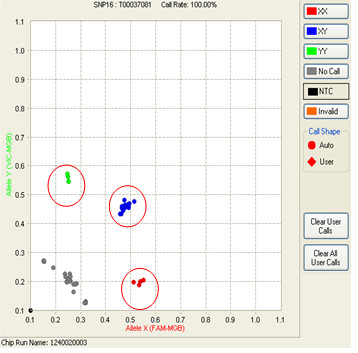
**Comparison of genotyping calls from the same sets of 15 samples that were either genotyped directly, or after the STA step**. The circled dots are data points generated from the STA samples.

When analyzing the call rate sample-by-sample, we noticed that 15 samples from the adenoma project contributed to over 50% of the total no call rate (failed to have valid genotypes). We amplified these 15 samples using a Specific Target Amplification (STA) protocol on the 47 targeted SNPs [24]. The amplified samples were diluted five- fold after STA and then genotyped on 48.48CS dynamic array. Figure 4 shows the genotyping results from both genomic and STA DNA from these 15 samples. The STA and genomic DNA were genotyped on the same chip side-by-side. The circled data points are from STA samples, which clearly showed much higher signal levels than the genomic DNA samples. With STA, all 15 samples except for one, had their genotype call-rate across 47 SNPs improve significantly, with most of them having call rates of up to 98 to 100% (Table 3). One sample could not be amplified, reflecting the possible presence of some form of PCR inhibitor in the original sample. The overall call rate for the 994 samples improved to 99.5% with the STA step.

**Table 3:** STA improves the call rate of DNA samples

Sample Name	No Call	XX	XY	YY	Grand Total	Call rate w/o STA	Call rate with STA
SB303208	6	9	19	13	47	87.2%	100.0%
SB303368	34	9	1	3	47	27.7%	97.9%
SB303440	12	12	11	12	47	74.5%	100.0%
SB303578	15	11	13	8	47	68.1%	97.9%
SB303579	16	10	11	10	47	66.0%	95.7%
SB303632	7	15	12	13	47	85.1%	97.9%
SB303637	5	16	15	11	47	89.4%	100.0%
SB303638	5	16	16	10	47	89.4%	89.4% *
SB303655	41	2	2	2	47	12.8%	89.4%
SB303880	10	1	33	3	47	78.7%	100.0%
SB303913	11	10	13	13	47	76.6%	100.0%
SB303918	5	16	16	10	47	89.4%	100.0%
SB303919	15	16	7	9	47	68.1%	100.0%
SB304064	23	12	3	9	47	51.1%	100.0%
SB304072	6	16	13	12	47	87.2%	100.0%

* This sample failed STA, because of possible PCR inhibition.

### Input DNA quantity affects genotyping accuracy

**Figure 5:**
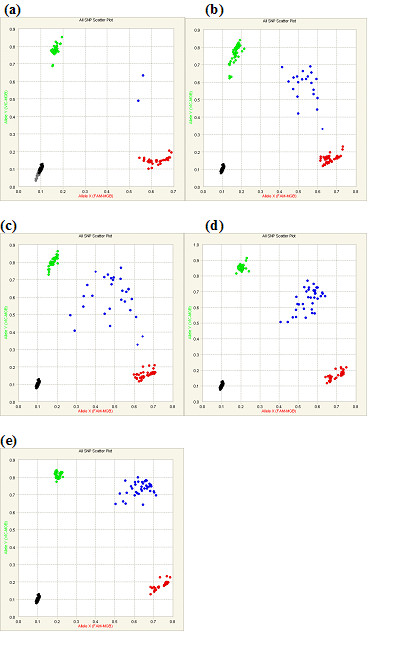
**Comparison of genotype call accuracy related to input DNA copy number**. Three genomic DNA samples carrying different genotypes with varied input amount were genotyped on SNP rs513349. The scatter plots of different DNA copy number ends in each reaction chambers are shown, **(a) **0.9 copies; **(b) **4.5 copies; **(c) **9 copies; **(d) **45 copies; **(e) **90 copies.

On the 48.48CS dynamic array, each DNA sample is distributed into 48 reaction chambers to genotype 48 SNPs. Typically, ~2 μl of DNA sample is made up to 5 μl with TaqMan^® ^Mastermix and other additives (see Materials and Methods), is loaded into the inlets of each chip, and is distributed into 48 reaction chambers, in a final volume of 6.5 nl per reaction chamber. When the initial number of DNA copies are low, as a result of the reduction in overall volume (approximately a 1000 fold reduction), the number of actual DNA molecules tested becomes limiting. In the experiments described earlier, each 6.5 nl reaction chamber contained about 25 copies of DNA molecules, in contrast to typical microtitre plates, which use about 500-600 fold more DNA. To evaluate the effect that DNA copy number has on genotyping calls, we tested three genomic DNA samples with three different genotypes for a single SNP (rs513349). Different amounts of DNA were loaded into Dynamic Array chips, such that the final DNA copy number in reaction chambers ranged from 0.9 to 90 copies of genomic equivalents. Figure 5 shows the genotyping clusters obtained with different amounts of input DNA.

As can be seen, when 45 to 90 copies of genomic DNA are tested, one gets excellent call rates (Table 4), with the heterozygotes called correctly in all cases. As the number of copies tested decrease, the numbers of no-calls and call errors increase. For example, when the copy number is 9 DNA molecules per reaction chamber, 22% (95% confidence interval: 0.16, 0.31) of the calls in the heterozygous cases failed to identify the correct genotype. Additionally, as would be predicted, when single molecules are tested, the heterozygotic calls (XY) decrease dramatically, with the number of homozygous calls and no-calls increasing proportionately. The tightness of the clusters (and therefore the call confidence) also decreases as the copy number decreases. Finally, when the DNA copy number averages 0.9 copies per reaction chamber, the no-call rate increases to 40%, 58% and 32% in the case of homozygotes A1A1, heterozygotes A1A2 and homozygotes A2A2, respectively. These no-call rates are significantly higher compared to those when the DNA copy number averages 4.5 copies per chamber (p value = 1.4E-17, 1.1E-31 and 2.3E-10, respectively). Meanwhile, the error rate of the calls in the heterogeneous case also increases significantly from 32% in the case of 4.5 copies per chamber to 84% in the case of 0.9 copies per chamber (p value:1.1E-14). This clearly shows the role that gene copy number and DNA concentration have on genotyping call quality and could have a significant impact on the data obtained from previous genotyping studies which have not monitored the amount of amplifiable DNA molecules in their sample preparation.

**Table 4:** Input DNA copy number and genotype call accuracy

			Copy number per chamber				
			
			0.9	4.5	9	45	90
**A1A1**	Genotype Count	No Call	50	2			
		
		YY	73	121	123	123	123
	
	Call rate		**59.3%**	98.4%	100%	100%	100%
	
	% Error		0%	0%	0%	0%	0%

**A1A2**	Genotype Count	No Call	72	4	7		
		
		XX	15	18	16		
		
		XY	8	80	89	123	123
		
		YY	28	21	11		
	
	Call rate		**41.5%**	96.7%	94.3%	100%	100%
	
	% Error		**35%**	**32%**	**22%**	0%	0%

**A2A2**	Genotype Count	No Call	39	4			
		
		XX	84	119	123	123	123
	
	Call rate		**68.3%**	96.7%	100%	100%	100%
	
	% Error		0%	0%	0%	0%	0%

**Grand No Call**			161	10	7	0	0

**Grand Error**			43	39	27	0	0

**Grand Call Rate**			56.4%	97.3%	98.1%	100%	100%

**Grand % Error**			11.7%	10.6%	7.3%	0.0%	0.0%

## Conclusion

In the current study we demonstrate the use of a unique nanofluidic genotyping system which is simple to use and permits medium multiplexing (tens to hundreds of SNPs) with high throughput, excellent call rates, call accuracy and low cost. We have demonstrated the use of the 48.48CS dynamic array with Integrated Fluidic Circuits (IFCs), by genotyping 994 individual human DNA samples on 47 different SNP assays, using nanoliter volumes of reagents. Calls from our platform were validated by selected genotyping of the same samples on the Applied Biosystems 7900 HT, while calls from the HapMap samples were compared with results obtained by the HapMap project.

Call rates of greater than 99.5% and call accuracy >99.8% were achieved from our study, which demonstrates that this is a formidable genotyping platform. The experimental set up is very simple, with a time-to-result for each sample of about 3 hours. In comparison, similar products by Illumina (GoldenGate ^® ^Assay) and Sequenom (iPlex™ assay) have reported call rates of only 99% and 90-95% respectively, take days (Illumina), not hours to obtain results, and have a complicated workflow(both Illumina and Sequenom) [25,26]. While we use TaqMan assays in our approach, the reduced reaction volume (6.5 nl) enables the cost of genotyping to be only 5 cents per data point, which compares very favorably with other platforms, which cost more, typically ranging by twice to an order of magnitude. While a detailed cost comparison is beyond the scope of this manuscript, it has been documented in other publications [27]. Thus, our approach has higher call rates, a significantly faster throughput, an easier workflow and lower cost than other medium throughput genotyping systems. The development of this nanofluidic genotyping system enhances the ability to screen mid-range numbers of SNPs across hundreds to thousands of samples.

## Methods

### Instrumentation and Nanofluidic Chips

The nanofluidic chip used in this study, 48.48CS dynamic array chips, the NanoFlex™ 4-IFC Controller and the BioMark™ Real-Time PCR System are manufactured by Fluidigm Corporation. The Nanoflex™ 4-IFC Controller utilizes pressure to control the valves in the chips and load samples and genotyping assay reagents into the reaction chambers. The BioMark system can be used to thermal cycle these nanofluidic chips and image the data in real time [22], and can also be used as an endpoint image reader. STA reactions were done in a GeneAmp PCR System 9700 from Applied Biosystems.

### TaqMan^® ^SNP Genotyping

For SNP genotyping on the dynamic array chips, a 5 μl sample mix was prepared for each sample containing 1× TaqMan^® ^Universal Master Mix (Applied Biosystems, Foster City, CA), 1 × GT Sample Loading Reagent (Fluidigm PN 85000741), 0.05 units/μl additional Taq-Gold polymerase (Applied Biosystems) and either 50~60 ng of genomic DNA, or diluted pre-amplified DNA. The TaqMan^® ^genotyping assays at 40 × were mixed with 1/2 volume of Dynamic Array (DA) assay loading reagent (Fluidigm PN 85000736) and 1/20 volume of 50 × ROX (Invitrogen) to make 10 × assay mixes (9 μM primers and 2 μM probe). Prior to loading the samples and assay mixes into the inlets, the chip was primed in the NanoFlex™ 4-IFC Controller. The 5 μl of sample mixes prepared as described were then loaded into each sample inlet of the dynamic array chip and 4 μl of 10 × genotyping assay mixes were loaded into assay inlets. The chip was then placed on the NanoFlex™ 4-IFC Controller for loading and mixing. After approximately 45 minutes the chip was ready for thermal cycling and detection of the reaction products on the modified stand-alone thermal cyclers. PCR was performed with an initial 2 min at 50°C and 10 min at 95°C, followed by 40 cycles of a 2-step amplification profile consisting of 15 s at 95°C for denaturation and 1 min at 60°C for annealing and extension. The endpoint fluorescent image data were acquired on the BioMark™ Real-Time PCR System. Data was analyzed using the Fluidigm SNP Genotyping Analysis software to obtain genotype calls.

### Human SNP Genotyping

We have used 48.48CS dynamic array chips to genotype 995 human DNA samples across 47 different SNP assays. A total of 89 HapMap Caucasian Trio samples and 905 Case: Controls from a Prostate, Lung, Colon, and Ovarian (PLCO) Cancer Screening Trial sponsored by the NCI, were genotyped in a blinded mode. A total of 2 μl of 25 ng/μl DNA from each sample were used in each sample reaction mix to genotype the 47 SNPs. These 995 samples were genotyped on 22 dynamic array chips. The reagents were prepared manually without using an automated robotic station. We used 3 stand-alone thermal cyclers and one BioMark™ Real-Time PCR System to perform PCR and read the chip runs on the single BioMark system after 40 cycles. Eight chips were run on each working day with a throughput of about 18,000 genotypes per day (48 samples × 47 assays × 8 chips). The endpoint chip reading was analyzed using the Fluidigm SNP Genotyping Analysis software.

### Specific Target Amplification

When either the quantity and/or the quality of the input DNA is not ideal, a Specific Target Amplification (STA) was performed to enrich targeted SNP sequences. The 40× TaqMan^® ^assays (with 36 μM primers) of targeted SNPs were mixed and diluted with DNA-free water to prepare 0.2 × assay mix (180 nM primers). STA was performed on a GeneAmp PCR 9700 system (Applied Biosystems, Foster City, CA) in a 5 μl reaction containing 2.5 μl of 2× TaqMan^® ^PreAmp master mix (Applied Biosystems, Foster City, CA), 1.25 μl of 0.2× of assay mix prepared as described and 1.25 μl of the DNA sample. Thermocycling conditions were 10 min at 95°C, followed by 14 cycles of a 2-step amplification profile of 15 sec at 95°C and 2 min at 60°C.

## Authors' contributions

RR, RW, RJ, and AC designed and organized the study. JW generated the experimental plan and performed data analysis. ML performed the dynamic array chip runs. RW, AC, AH, BH, MY, SB, WH, RH and JC designed and performed experiments on microtiter plates and compared results from both platforms. RW and AC proposed analyses and RW, AC, JW and RR interpreted the results. JW, RJ and RR wrote the manuscript.
